# Knowledge and caring

**DOI:** 10.1308/rcsann.2025.0007

**Published:** 2025-02-01

**Authors:** B Rogers

**Figure:**
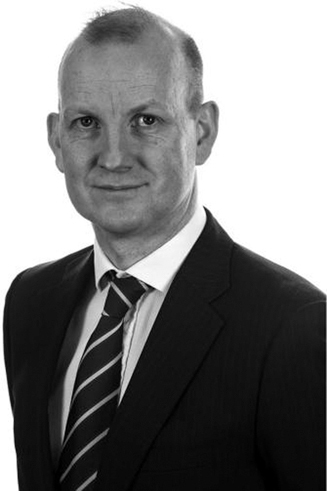


The *Annals* occupy a unique position in the field of medical publishing due principally to its heritage and that of the Royal College of Surgeons of England, an institution established 225 years ago. This is a source of both responsibility and pride to all staff, both paid and unpaid, that work on its production. However, reading the table of contents of the February 2025 issue provides a *prime facie* demonstration of its primary purpose: to disseminate novel and clinically relevant surgical research spanning specialties. The studies are broad in subject but relevant to the surgical provision in any hospital.

Khan *et al*^1^ analyse the opportunities to improve the management of acute colonic pseudo-obstruction, a potentially fatal condition with an incidence of 1:1000 inpatient stays per year. This review and its outcomes are relevant to all clinicians with responsibility for inpatients.

Therapeutic mammaplasty affords large breast tumour resections with optimal aesthetic outcomes but risks higher wound complications which often necessitate delaying adjuvant therapy. Rampal *et al* evaluate this clinical decision in oncoplastic breast conservation surgery.^2^

Where should laparoscopic cholecystectomy in children be performed; adult or paediatric centres? Such regional level data are vital to the planning of both service provision and training. Sinha *et al*. provide a meta-analysis of 92 studies, over 74,000 procedures, analysing this clinical question.^3^ Continuing the theme of paediatric surgery, how frequently does a trainee act as a trainer in laparoscopic appendicectomy? Henderson *et al* present how trainees contribute to intra-operative teaching, an essential surgical skill.^4^ Furthermore, the question of valid consent, particularly the provisional of information on reasonable alternatives, for the treatment of acute appendicitis is considered by Bethell *et al.*^5^

The unstable trauma patient features in the published studies in this edition. Maya *et al* evaluate the timing of emergency laparotomy surgery in unstable isolated abdominal trauma patients and suggest that optimal use of time *before* surgery may afford beneficial outcomes.^6^ Civilian gunshot injuries are unfortunately seen in all countries, and a registry-based retrospective study from Brazil demonstrates that the non-operative management is a valid option in selected patients.^7^

The above short descriptions highlight the diversity of topics the *Annals* can publish and provide thought for surgeons across all specialties. This journal's strength is its ability to attract such varied but high-quality studies.

I recently attended hospital as the next of kin of a family member and witnessed excellent caring and empathy from the whole surgical team. This highlighted the importance of holistic care. As surgeons, we must concurrently evaluate clinical advances such as those published in the *Annals* while striving to provide empathic care to often vulnerable and frightened patients.
